# Tunable Electronic and Optical Properties of Al- and Fe-Doped Lizardite/h-BN Heterostructures

**DOI:** 10.1021/acsomega.4c07584

**Published:** 2024-11-07

**Authors:** Angsula Ghosh, Puspitapallab Chaudhuri, C. A. Frota, Hidembergue Ordozgoith da Frota

**Affiliations:** † Department of Materials Physics, Federal University of Amazonas, 69077-000 Manaus, AM, Brazil; ‡ Department of Civil Engineering, Federal University of Amazonas, 69077-000 Manaus, AM, Brazil

## Abstract

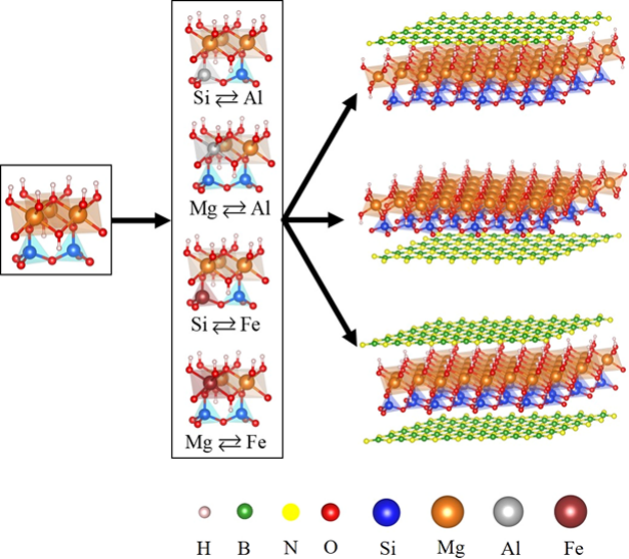

Recent advancements in the chemical substitution of clay minerals have yielded promising results in the development of innovative materials. The utilization of the serpentine mineral lizardite holds significance not only due to its abundance in nature but also for its environmentally friendly characteristics. A comprehensive investigation of a lizardite/h-BN van der Waals heterostructure in the presence of impurities has been conducted. Formation energy calculations for the 12 possible heterostructures demonstrate the chemical stability of all configurations. The analysis of energy bands and density of states reveals the altered electronic properties of the system attributed to impurities. Substituting Al for Mg and Si induces a transition to a metallic state, whereas characteristics due to Fe substitution depend on its position. Substituting Fe for Mg results in a metallic nature, while substitution for Si maintains semiconducting behavior with a reduced band gap compared to the pristine case. Furthermore, the modified optical properties of the heterostructures broaden the potential applications of h-BN and the clay mineral, leading to significant advancements in optoelectronic and field-effect devices.

## Introduction

The advent of graphene as a multifunctional material has ushered in a new era of possibilities for 2D materials.^1^ Among these, hexagonal boron nitride (h-BN) draws significant attention not only due to its exceptional thermal and chemical stability but also due to its distinct dielectric properties.^2,3^ Its versatility has led to a wide range of applications mainly due to a combination of unique properties like good electrical insulation, excellent lubricating properties, and environment friendliness.^3−5^ However, the large band gap of h-BN has limited its utility in nanoelectronic devices. Consequently, heterostructures consisting of layers of h-BN combined with 2D materials such as graphene, transition metal dichalcogenides (MoS_2_, WS_2_), and silicene have been extensively studied due to their potential applications across various fields.^6−53^ Vertical stacking with a layered architecture has effectively developed a 2D nanotransistor incorporating h-BN, graphene, and MoS_2_.^9^

Lizardite [Mg_3_(Si_2_O_5_)(OH)_4_], probably the most abundant serpentine mineral, is a Mg-rich layered clay mineral.^10^ Unlike other minerals in the serpentine group, lizardite demonstrates an ideal layer topology due to the shifts of the octahedral and tetrahedral cations away from their ideal positions and to the limited Al^3+^ for Si^4+^ substitution in tetrahedral sites.^11^ It is not only cheap and widely available in nature but also environmentally and economically friendly. Additionally, it stands as the most stable polymorph under ambient conditions, undergoing transformation to antigorite at elevated pressures and temperatures.^12,13^ Furthermore, the clay mineral may undergo chemical and mechanical alterations due to the presence of defects or substitutional impurities such as Al, Fe, Mn, and Co, among others, offering avenues for the development of novel materials with unique properties.^14^ Amid these impurities, Fe and Al are prevalent in lizardite and have recently been the subject of studies aiming to comprehend the alterations in vibrational properties^15^ and the structural composition^16^ induced by these impurities. Additionally, the substitution of Al and Fe for Mg and Si leads to stronger interactions between adjacent layers, thereby enhancing stability.^10^ Low-cost production of 2D materials shows intriguing properties^17−19^ compared to their bulk counterparts, which inspired us to consider the layered form of the serpentine mineral. Furthermore, a recent experimental study using scanning probe microscopy demonstrated that thenatural serpentine mineral can be mechanically exfoliated, yielding well-characterized 2D serpentine flakes.^19^ This finding highlighted serpentine’s potential as an alternate source of a low-cost two-dimensional (2D) nanomaterial. Moreover, elemental substitution (e.g., by Al and Fe) in monolayer vermiculite provides evidence of the possible origin of the inherent electric dipole and resultant ferroelectricity.^17^

Recent developments in van der Waals heterostructures have led to studies on doping, with potential applications in the semiconductor electronics industry due to the high mobility and carrier concentration. Various doping techniques have proven successful in 2D materials such as electrolyte gating and chemical intercalation,^20−22^ molecular self-assembling on surfaces,^23,24^ photoinduced doping,^25,26^ and electron beam radiation using a scanning electron microscope.^27−29^ However, some doping methods could considerably deteriorate conductivity and alter the interface at 2D heterostructures.^30^ Hence, it is important to explore alternative approaches to achieve high-quality 2D van der Waals structures. Recently, the electronic properties of graphene stacked on talc crystal^30^ whose structure resembles the mineral lizardite were considered involving impurities such as Al and Fe. Hence, inspired by the stability of h-BN as an ideal substrate for various materials and considering the possibility of the presence of impurities in 2D lizardite, we have systematically explored the electronic structure and properties of two insulating materials in a vertical architecture.

In this work, we performed a detailed study to explore how the electronic properties of lizardite/h-BN heterostructures are influenced by the introduction of Al and Fe in place of Mg and Si. Density functional theory has been utilized for the study. The optical properties have also been investigated in all of the architectures chosen. The computational method and the relevant technical details are detailed in the next section, whereas the results and discussion on the doped heterostructures in the presence of impurities are provided in the later section. The last section provides a brief summary and conclusion on the work.

## Calculation Details

The electronic and structural properties of lizardite/h-BN vertical heterostructures were investigated, incorporating impurities (Al or Fe) in lieu of Mg and Si arranged in three diverse configurations. This study considers the influence of doping on the 12 van der Waals heterostructures formed, depending on the architecture and also the type and position of the impurities. The Quantum Espresso code (QE) was utilized in all calculations using periodic boundary conditions^31^ in the density functional approach. Ultrasoft pseudopotential (USPP)^32^ was utilized with a generalized gradient approximation (GGA) for the exchange–correlation energy functional PBEsol.^33^ Additionally, corrections for van der Waals interaction were incorporated using semiempirical Grimme’s DFT-D2^34,35^ proposal. The kinetic energy and the charge density cutoffs for the plane wave functions are 0.8 and 8 keV, respectively. Brillouin zone was sampled using a Monkhorst–Pack^36^ grid, where a 12 × 12 × 1 grid mesh^36^ was utilized for the supercell. All of the systems were fully optimized to obtain the crystal structures and the electronic properties of the systems. For the optimization procedures, we utilize the convergence thresholds on forces and total energy to be 10^–6^ eV/Å and 10^–5^ eV, respectively. Xcrysden^37^ and Vesta^38^ packages were employed for the visualization of the heterostructure structures. The formation energy *E*_f_^d^ of the doped structures was calculated using eq 1, which is given by
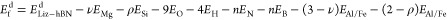
1where *E*_Liz–hBN_^d^ is the energy of doped lizardite/h-BN, *E*_X_ is the energy of the constituent X, and *n* is the number of boron/nitrogen atoms in h-BN layers. *n* = 4 in heterostructures Liz-hBN1 and Liz-hBN2, whereas *n* = 8 in Liz-hBN3. Depending on the type of doping, ν and ρ determine the number of atoms of Mg and Si, respectively. For the four types of substitutions, we have Al_Si_, ν = 3 and ρ = 1; Al_Mg_, ν = 2 and ρ = 2; Fe_Si_, ν = 3 and ρ = 1; and Fe_Mg_, ν = 2 and ρ = 2. Furthermore, we successfully computed the binding energy *E*_b_ of the doped heterostructures by considering the relaxed structures of the constituent layers, which can be expressed as
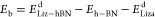
2where *E*_h-BN_ and *E*_Liza_^d^ are the energies of the h-BN layers and doped lizardite slabs, respectively. After the structural optimizations, the electronic properties of all fully optimized structures were analyzed based on the density of states, energy bands, and also the projected density of states for all of the elements of the lizardite/h-BN with impurities. On the other hand, it is known that the DFT method tends to underestimate the band gap values. Hence, incorporation of the Heyd–Scuseria–Ernzerhof hybrid functional (HSE) approach^39^ is crucial for obtaining a more accurate understanding of the gap in the heterostructures exhibiting semiconducting characteristics. The mixing parameter, α (typical value), used for the HSE calculation is 0.25.

The optical properties of the 12 architectures were determined using epsilon.x, a post-processing code of PWSCF. It is important to note that norm-conserving pseudopotentials were considered for the calculations of reflectivity, absorption coefficient, energy loss spectrum, and refractive indices utilizing exchange–correlation functionals implemented within the generalized gradient approximation (GGA).^40^ The properties were compared with those of pristine heterostructures and also a few other materials found in the literature.

## Results and Discussion

**Figure 1:**
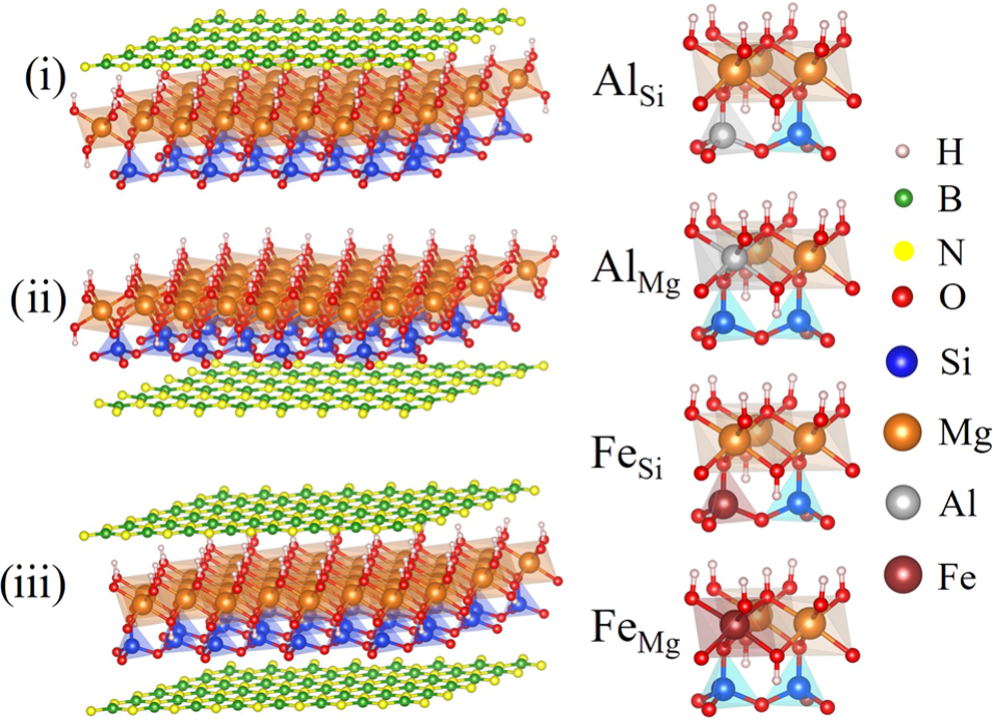
Lizardite/h-BN-optimized structures: (i) a sheet of h-BN on top of the octahedral layer, (ii) a h-BN sheet below the tetrahedral layer, and (iii) two h-BN sheets simultaneously above the octahedral layer and below the tetrahedral layers. The substituted lizardite utilized in the lizardite/h-BN structures is Al in place of Si (Al_Si_), Al in place of Mg (Al_Mg_), Fe in place of Si (Fe_Si_), and Fe in place of Mg (Fe_Mg_).

In this work, a comprehensive study of the effect of substitution on the electronic and structural properties of lizardite/h-BN heterostructures has been performed. Figure 1 demonstrates the optimized structures of lizardite/h-BN in the presence of impurities along the [001] direction: (i) a h-BN sheet on the top of the octahedral lizardite layer (Liz-hBN1), (ii) a h-BN sheet below the tetrahedral lizardite layer (Liz-hBN2), and (iii) two h-BN sheets simultaneously above the octahedral and below the tetrahedral layers (Liz-hBN3). The above figure was created using the Vesta^38^ software package. The substituted lizardites utilized in the lizardite/h-BN structures are Al_Si_ Al_Mg_ Fe_Si_ and Fe_Mg_. Hence, there are a total of 12 different heterostructures of type X_Y_, where X can be Al or Fe and Y can be Si or Mg. The Mg atom is indicated in orange, Si in blue, hydrogen in white, oxygen in red, Al in gray, Fe in maroon, B atoms in green, and N atoms in yellow. The supercells considered for the single layer of h-BN above/below the doped lizardite consist of 26 atoms, whereas that for the layers of h-BN on top of the octahedral layer and below the tetrahedral layer of the doped lizardite layer simultaneously contains 34 atoms. The optimized distance between the h-BN plane and the oxygen plane of lizardite in all 12 configurations is exhibited in Table 1. All four configurations of Liz-hBN1 exhibit a decrease in the distance between h-BN and the oxygen plane of the lizardite slab compared to that of the corresponding pristine heterostructure, which is 3.16 Å. However, for those of Liz-hBN2 and Liz-hBN3, we do not observe such uniform modifications. In the case of Liz-hBN2, a decrease is observed for X_*Si*_, whereas for Liz-hBN3, the distances *d*_1_ decrease for Al_Mg_ and increase for Al_Si_. For Fe_*Y*_, *d*_1_ remains unaltered. Considering *d*_2_ for Liz-hBN3, we observe an increase for X_Mg_ and a decrease for X_Si_. The highest modification occurs for Al_Mg_ in Liz-hBN1 and Al_Si_ in Liz-hBN2. The same trait is also observed for Liz-hBN3. *d*_1_ is most modified for Al_Mg_, whereas *d*_2_ changes most for Al_Si_. *d*_2_ is 3.05 Å in pristine Liz-hBN2, whereas in pristine Liz-hBN3, *d*_1_ and *d*_2_ are 3.14 and 3.03 Å, respectively. Due to the difference in the electronegativity of the impurity atoms compared to those of the substituted ones, the bond lengths also suffer alterations. The Si atom has a higher electronegativity than both Al and Fe, which leads to an increase of the Al–O and Fe–O bond lengths than Si–O. On the other hand, Mg has a lower negativity than Al and Fe; hence, the substitution of Mg by Al and Fe leads to a decrease in the Al–O and Fe–O bond lengths.

**Table 1:** Structural Data and the Formation and Binding Energies of the Heterostructures

	Liz-hBN1			Liz-hBN2			Liz-hBN3			
doping	*d*_1_ (Å)	*E*_f_^d^ (Ry)	*E*_b_ (eV)	*d*_2_ (Å)	*E*_f_^d^ (Ry)	*E*_b_ (eV)	*d*_1_ (Å)	*d*_2_ (Å)	*E*_f_^d^ (Ry)	*E*_b_ (eV)
Al_Si_	3.16	–13.46	–0.54	2.76	–13.47	–0.84	3.17	2.79	–18.75	–1.45
Al_Mg_	2.84	–13.81	–0.50	3.10	–13.80	–0.36	2.83	3.14	–19.07	–0.80
Fe_Si_	3.14	–13.52	–0.55	3.00	–13.51	–0.39	3.15	3.00	–18.79	–1.02
Fe_Mg_	3.13	–13.95	–0.57	3.04	–13.94	–0.37	3.15	3.06	–19.22	–0.99

The formation (*E*_f_^d^) and binding (*E*_b_) energies for the 12 compositions are exhibited in Table 1. The negative formation energies and the binding energies of the systems demonstrate the possibility of formation of doped heterostructures. Among the four possible formations in the three types of stacking, Fe_Mg_ has the highest possibility of formation, whereas the least is for Al_Si_, demonstrating a clear dependence on the type of impurity. In the case of the binding energy from the layers, Al_Si_ has the lowest binding energy in Liz-hBN2 and Liz-hBN3. For Liz-hBN1, it occurs for Fe_Mg_. For Liz-hBN2 and Liz-hBN3, *E*_b_ can be arranged as follows: Al_Si_ < Fe_Si_ < Fe_Mg_ < Al_Mg_. In Liz-hBN1, the above sequence is Fe_Mg_ < Fe_Si_ < Al_Si_ < Al_Mg_. Hence, irrespective of the value, *E*_b_ illustrates the cohesion of the layers to form heterostructures from lizardite with impurity and h-BN layers.

**Figure 2:**
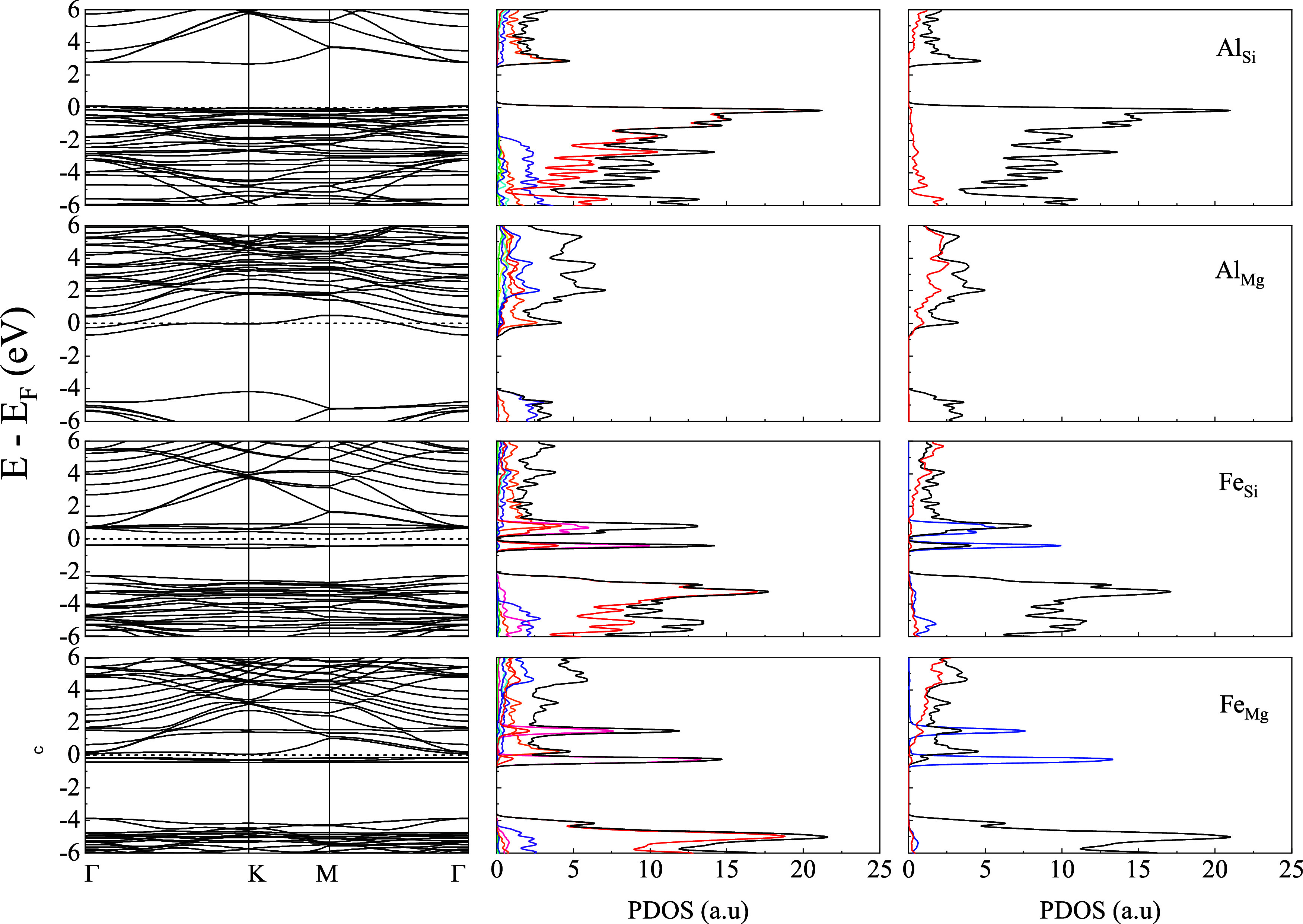
Energy bands (column 1), the projected density of states of the atoms (column 2), and the orbital-resolved PDOS (column 3) of Liz-hBN1 in the presence of impurities Al/Fe for the cases of Al_Si_, Al_Mg_, Fe_Si_, and Fe_Mg_ as indicated in the figure. Column 2 shows the contributions from Mg (green), Si (blue), O (red), H (cyan), B (orange), N (violet), Al (yellow), and Fe (violet), whereas column 3 has the contributions from s-wave (red line), p-wave (black line), and d-wave (blue line). The Fermi energy is shown by the dotted line in the first column of the figure.

**Figure 3:**
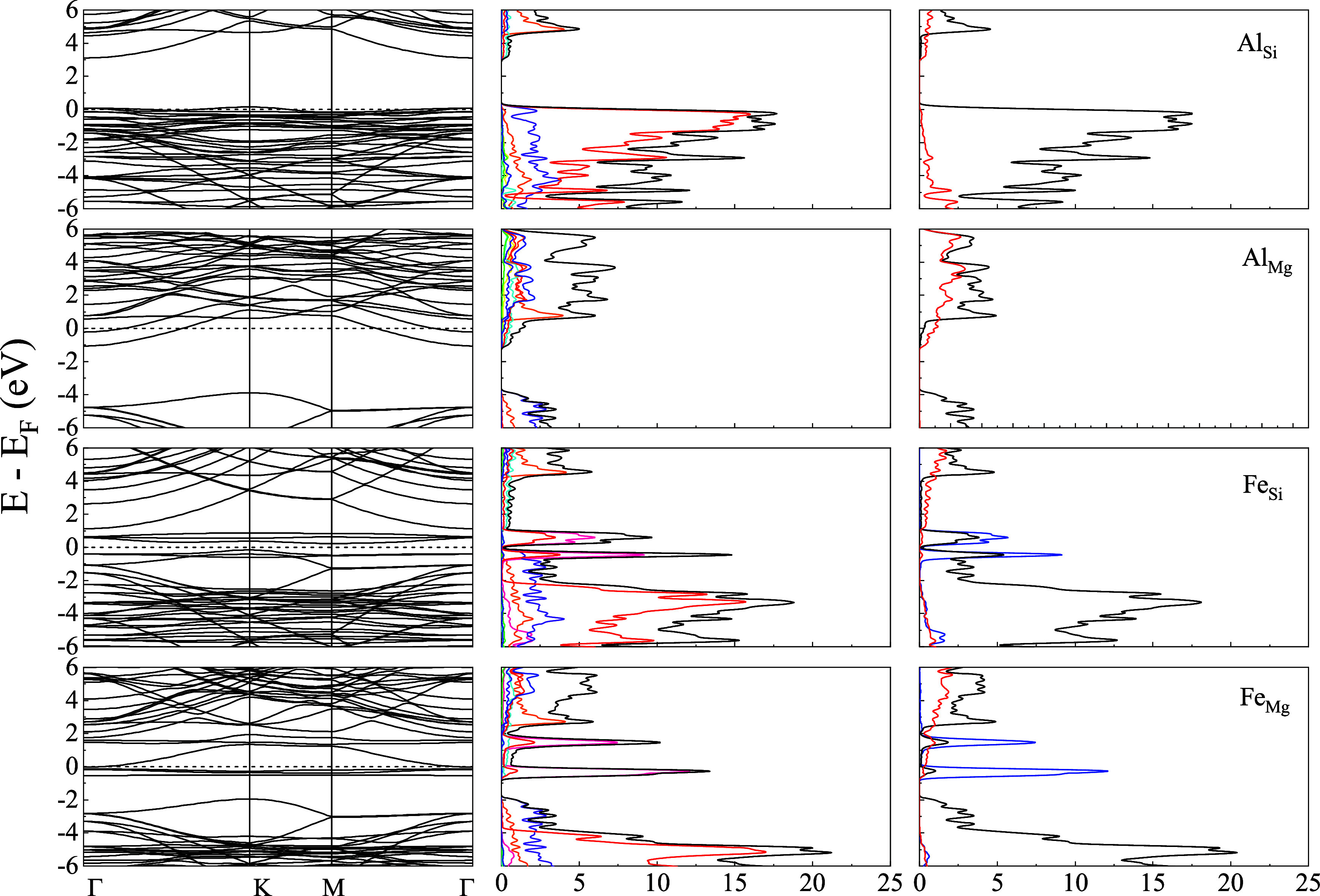
Energy bands (column 1), the projected density of states of the atoms (column 2), and the orbital-resolved PDOS (column 3) of Liz-hBN2 in the presence of impurities Al/Fe for the cases of Al_Si_, Al_Mg_, Fe_Si_, and Fe_Mg_ as indicated in the figure. Column 2 shows the contributions from Mg (green), Si (blue), O (red), H (cyan), B (orange), N (violet), Al (yellow), and Fe (violet), whereas column 3 has the contributions from s-wave (red line), p-wave (black line), and d-wave (blue line). The Fermi energy is shown by the dotted line in the first column of the figure.

**Figure 4:**
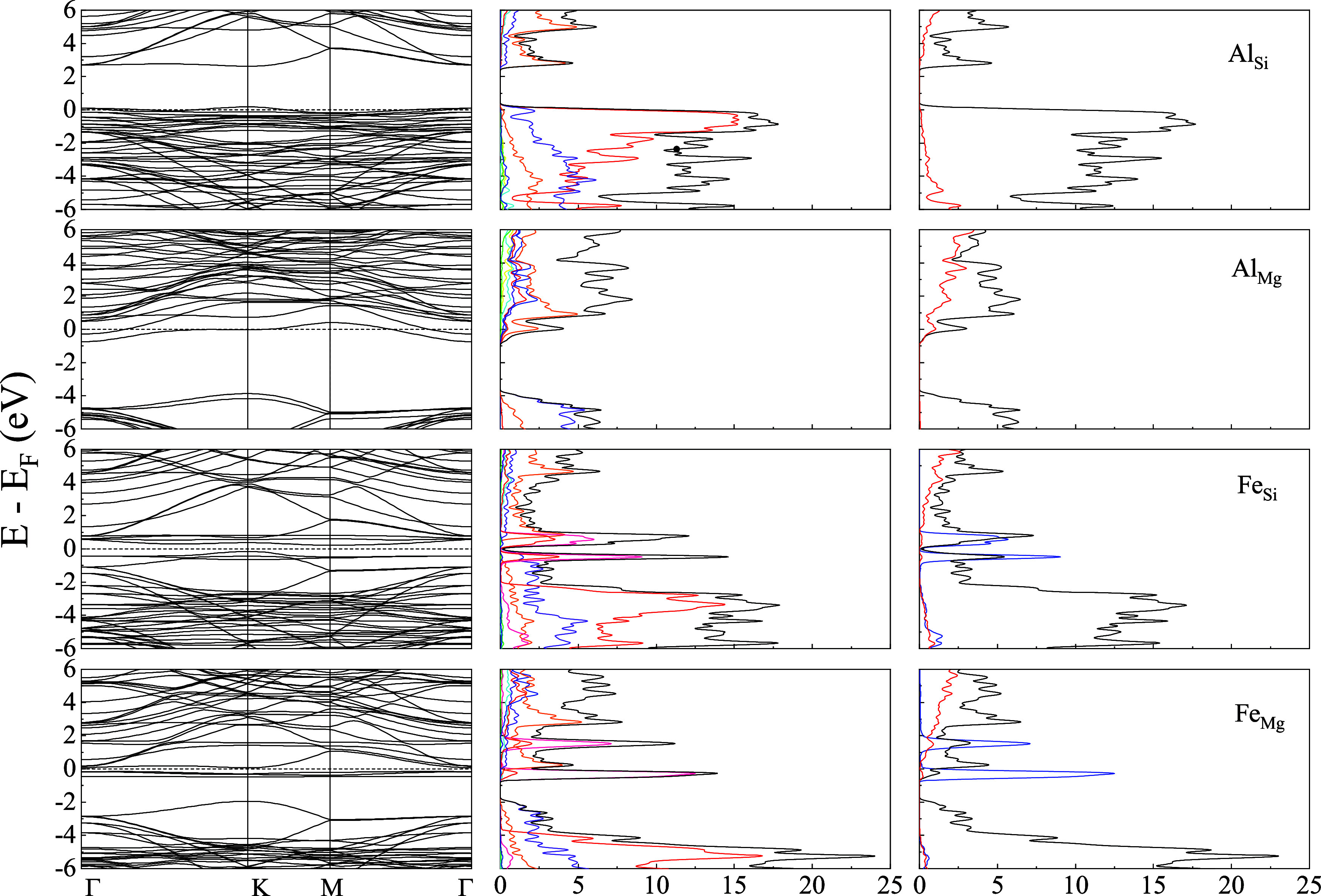
Energy bands (column 1), the projected density of states of the atoms (column 2), and the orbital-resolved PDOS (column 3) of Liz-hBN3 in the presence of impurities Al/Fe for the cases of Al_Si_, Al_Mg_, Fe_Si_, and Fe_Mg_ as represented in the figures. Column 2 shows the contributions from Mg (green), Si (blue), O (red), H (cyan), B (orange), N (violet), Al (yellow), and Fe (violet), whereas column 3 has the contributions from s-wave (red line), p-wave (black line), and d-wave (blue line). The Fermi energy is shown by the dotted line in the first column of the figure.

Figures 2–4 present the energy band structure, projected density of states (PDOS), and the orbital-resolved PDOS due to the presence of impurities Al/Fe in place of Mg and Si in Liz-hBN1, Liz-hBN2, and Liz-hBN3, respectively. The four systems, Al_Si_, Al_Mg_, Fe_Si_, and Fe_Mg_, are shown in the figures. It should be essential to comprehend the modifications in the electronic properties of the heterostructures due to the presence of impurities in lizardite. The path utilized for the plot of the energy bands is given by Γ–K–M−Γ. The band structures and the PDOS of the pristine lizardite slab and the pristine lizardite/h-BN heterostructures are in complete agreement with those in earlier studies.^14,41^ The contributions from Mg (green), Si (blue), O (red), H (cyan), B (orange), N (violet), Al (yellow), and Fe (violet) are exhibited in the second column of the figures. The orbital-resolved PDOS are displayed in the last column with s-wave (red line), p-wave (black line), and d-wave (blue line) contributions.

The energy bands and PDOS demonstrate a clear charge transfer between the impurities in lizardite and the h-BN states. The substitutional impurities in Liza/h-BN heterostructures lead to a transition to a metallic state for Al_Mg_, Al_Si_, and Fe_Mg_ in Liz-hBN1, Liz-hBN2, and Liz-hBN3. However, the heterostructure Fe_Si_ continues to demonstrate the semiconducting characteristics with energy gaps of 0.65, 0.37, and 0.36 eV in Liz-hBN1, Liz-hBN2, and Liz-hBN3, respectively. The pristine Liz-hBN1, Liz-hBN2, and Liz-hBN3 heterostructures have energy gaps of 2.76, 1.58, and 1.08 eV, respectively,^41^ whereas lizardite and h-BN are insulators with the calculated gaps of 3.64 and 6.0 eV, respectively. The 0.65 eV gap closely resembles the energy gap of semiconductors, which are important in the technological industry. Analyzing the PDOS of doped Liz-hBN1, we observe that similar to pristine Liz-hBN1, the contribution from the O atom continues to dominate the valence band for all of the cases except Al_Mg_. Moreover, PDOS in the case of Al_Mg_ is also much less than the other three substituted heterostructures. The contribution from the Fe atom is considerably high close to the Fermi energy. In the case of the orbital-resolved PDOS, *p*-wave dominates for all of the cases. The *d*-wave contribution from the Fe atom is exhibited at energies very close to the Fermi energy for the Fe_Y_ systems. In order to understand the changes brought about by doping, we consider the characteristics of the impurity atoms and also the substituted atoms. Among the four atoms, Fe is significantly larger and heavier than Al, Mg, and Si. Additionally, the 3*d* orbital of Fe plays an important role in these modifications. In the case of Al_Y_, we observe minimal changes in the structure of the energy bands and PDOS compared to the pristine counterparts, where Al acts as a donor impurity in Al_Mg_ and as an acceptor in Al_Si_. In the case of Fe_Y_ systems, however, Fe plays a very different role and modifies the band structure profoundly. The creation of an energy level near the Fermi energy due to Fe alters the energy bands, thereby modifying the system’s electronic structure.

In order to address the energy band gap of the heterostructures, we calculate the optimized structures of the semiconducting heterostructures utilizing the HSE formalism. For pure insulating lizardite, the energy band gap is 5.18 eV, whereas the Fe_Si_ structures demonstrate gaps of 3.90, 2.02, and 1.99 eV in Liz-hBN1, Liz-hBN2, and Liz-hBN3, respectively. Hence, a decrease in the energy band gap is evident for all of the Fe_Si_ structures. Notably, the gap is highest for Liza-hBN1 and lowest for Liza-hBN3, as observed before.

The presence of the impurities also induces a shift in the Fermi energy of the systems, as expected due to doping. An upshift is observed for those exhibiting n-type doping and a downshift for those with p-type doping. X_Mg_ shows an upshift in all of the three architectures chosen to form the heterostructures. On the contrary, X_Si_ exhibits a downshift for the heterostructures. The highest shift for the Fermi energy also occurs for Al_Si_ and is equal to 3.06, 2.64, and 2.21 eV in Liz-hBN1, Liz-hBN2, and Liz-hBN3, respectively. The least affected region is that of Fe_Mg_.

To enable us to understand the doped heterostructures and also compare them with the pristine heterostructures, we considered the optical properties of the systems. A detailed study of the absorption coefficient, electron energy loss spectra (EELS), reflectivity, and refractive index are performed. These properties are evaluated by using the complex dielectric tensor as a function of incident electromagnetic radiation frequency, where the subscripts denote polarization vector components. The random phase approximation method was utilized along with the Kohn–Sham eigenstates and eigenvalues derived from DFT calculations to calculate the imaginary part of the dielectric function. The real part was determined from the imaginary part of the dielectric function using the Kramers–Kronig relation.^42^

**Figure 5:**
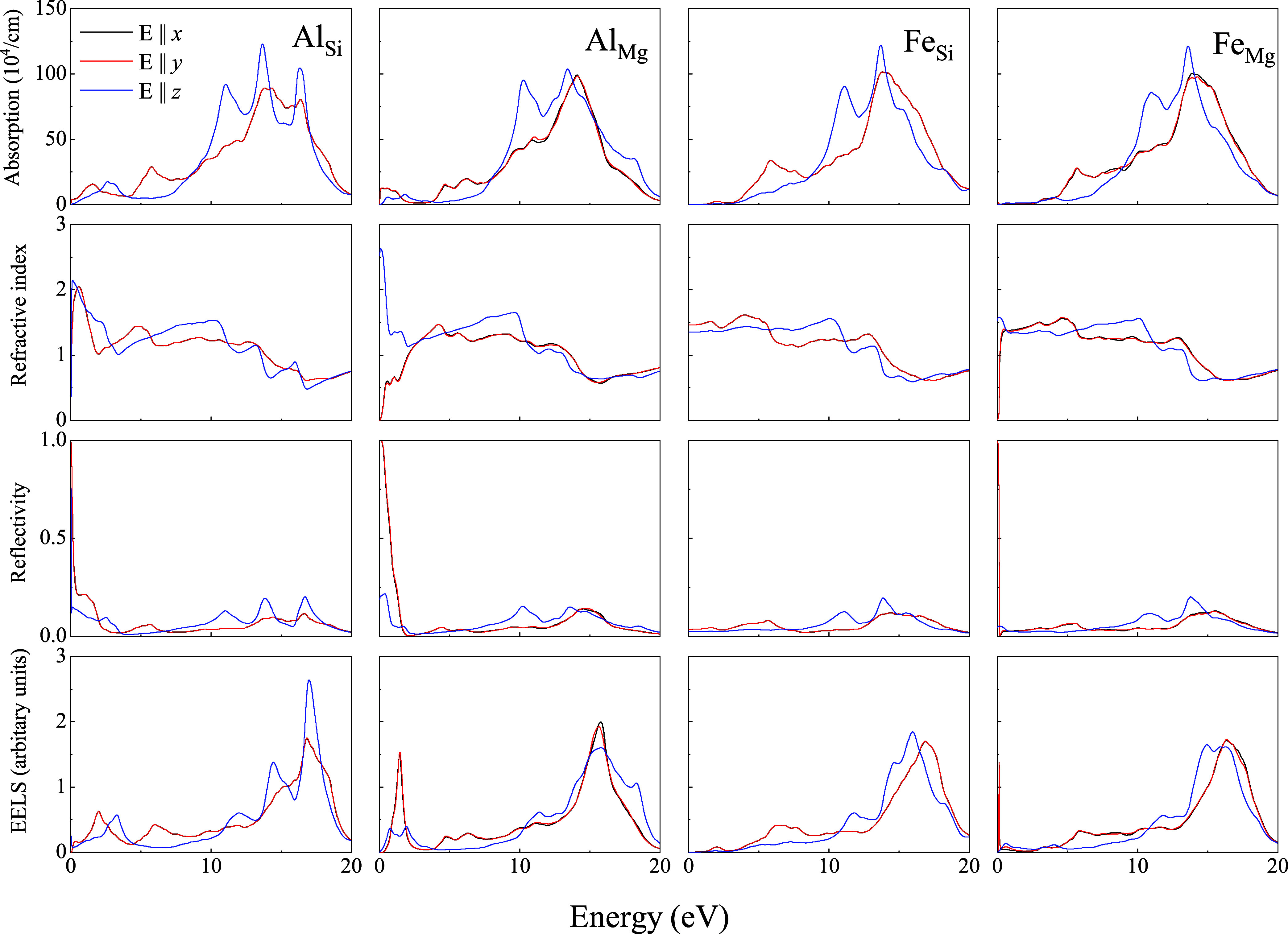
Absorption coefficients (first row), refractive index (second row), reflectivity (third row), and electron energy loss (fourth row) of Liz-hBN1—Al_Si_, Al_Mg_, Fe_Si_, and Fe_Mg_ as represented in the figures.

**Figure 6:**
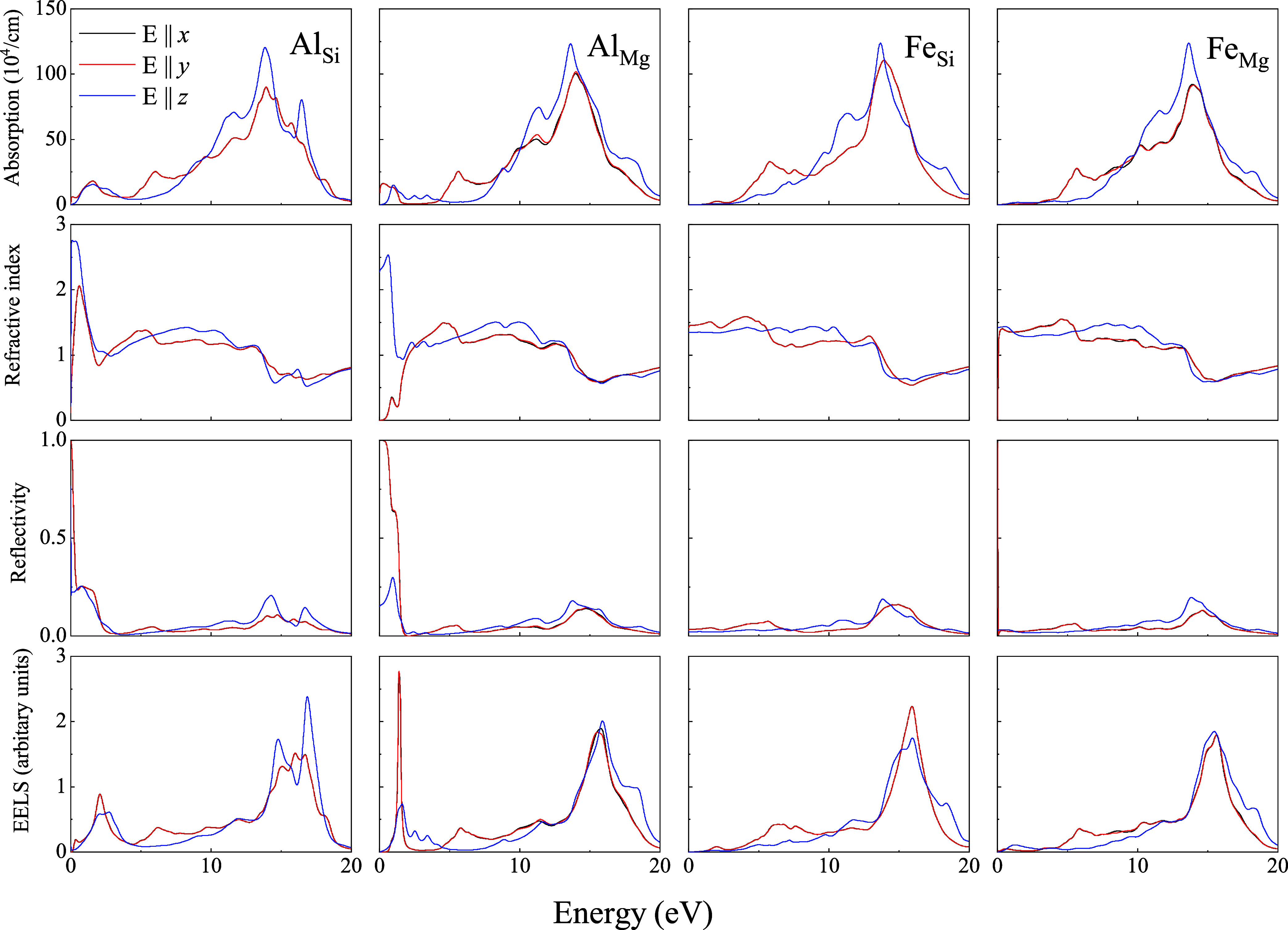
Absorption coefficients (first row), refractive index (second row), reflectivity (third row), and electron energy loss (fourth row) of Liz-hBN2—Al_Si_, Al_Mg_, Fe_Si_, and Fe_Mg_ as represented in the figures.

**Figure 7:**
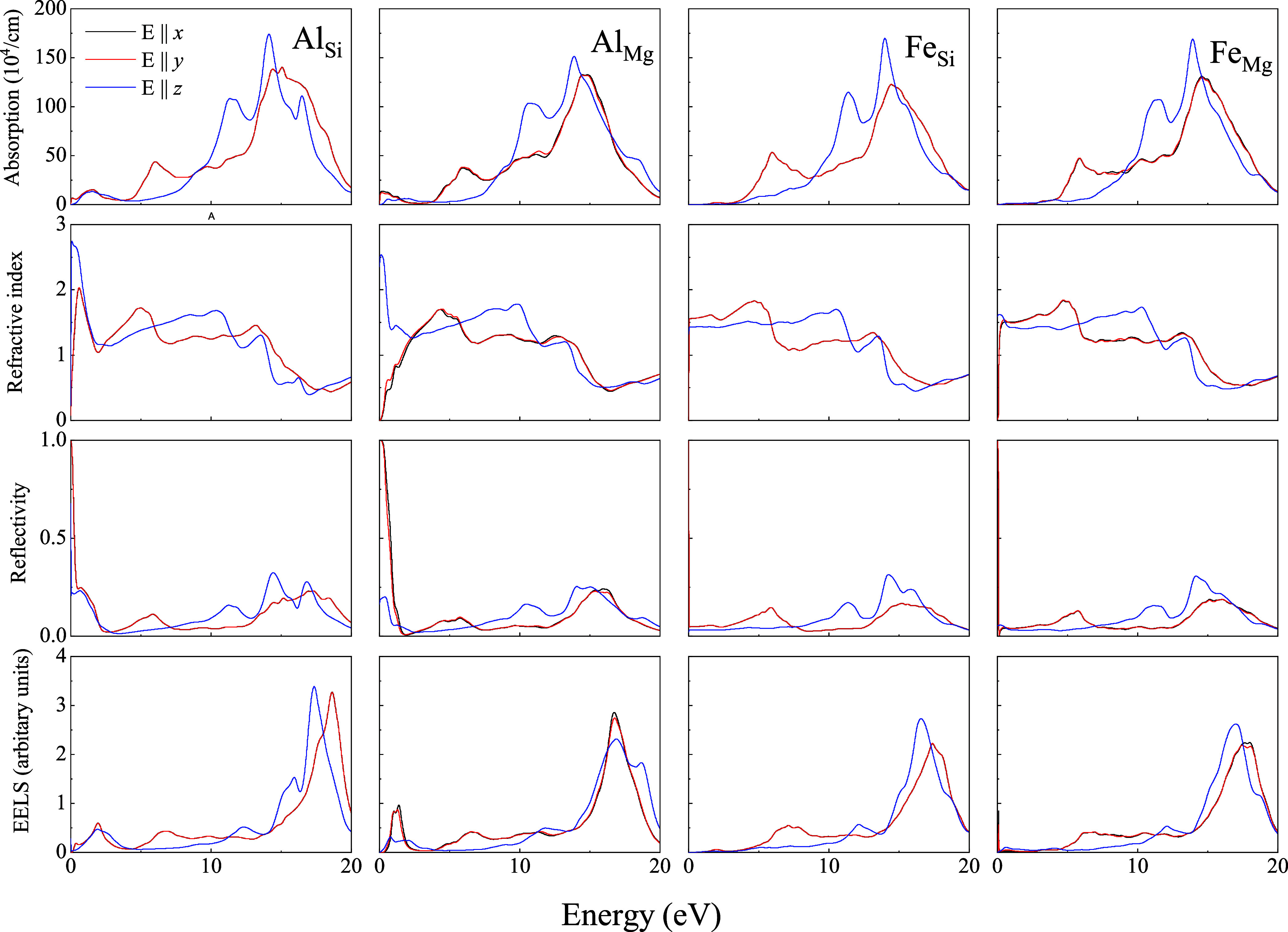
Absorption coefficients (first row), refractive index (second row), reflectivity (third row), and electron energy loss (fourth row) of Liz-hBN3—Al_Si_, Al_Mg_, Fe_Si_, and Fe_Mg_ as represented in the figures.

Figures 5–7 display the absorption spectra (first row), refractive index (second row), reflectivity (third row), and electron energy loss (fourth row) of Liz-hBN1, Liz-hBN2, and Liz-hBN3, respectively, in the range between 0 and 20 eV, which encompasses the infrared, visible, and ultraviolet ranges of the electromagnetic radiation spectrum. The optical properties of the doped lizardite/h-BN heterostructures Al_Si_, Al_Mg_, Fe_Si_, and Fe_Mg_ are exhibited in the first, second, third, and fourth columns, respectively. For in-plane polarization (**E** ∥ *x* and **E** ∥ *y*), the optical properties are denoted in red, whereas those for the polarization direction normal to the layer (**E** ∥ *z*) are represented in blue.

In Figures 5–7, the absorption coefficients for the in-plane polarization and perpendicular to the layer in doped heterostructures demonstrate characteristics that are similar to those of the pristine architectures.^41^ The peaks at around 6 and 13.5 eV prevail in all of the substituted heterostructures for in-plane polarization. Due to the disappearance of the gap and the transition to the metallic state, unlike the pristine heterostructures, the absorption coefficient is nonzero in the infrared and visible spectrum. Moreover the coefficients in the above regions are more prominent in the Al_Y_ systems than those in Fe_Y_. The presence of a small gap in Fe_Si_ is also evident. Using HSE calculations, the gaps in the above systems are in agreement with those in the electronic calculations. For **E** ∥ *z*, two peaks are observed in the ultraviolet region for all substituted heterostructures considered in this work, except in Al_Si_ architectures, where we observe three peaks in the same region of the spectrum. In Al_Si_ structures, a third peak at around 17 eV is observed, as present in the pristine lizardite slab.^41^

Refractive indices demonstrated in the third row of Figures 5–7 exhibit modifications primarily in the infrared and visible spectrum of Al_Y_ systems compared to the pristine counterparts.^41^ Refractive indices of Fe_Y_ of all three heterostructures continue to be similar to those of the pristine ones. Al_Y_ systems exhibit increased indices in the infrared and visible region for **E** ∥ *z*. However, for in-plane polarization, the refractive index is lower in the infrared region of the spectrum and increases with an increase in the energy values. The anisotropy in the static refractive indices (value at zero energies) increases compared to the pristine lizardite slab and h-BN layers. For Al_Y_ systems, they are around 2.5, whereas for Fe_Y_, they are approximately 1.5. The index for the lizardite slab is 1.20 in both directions, whereas those of h-BN are 1.21 and 1.37 for in-plane and perpendicular directions of polarizations, respectively. It is important to note that clay minerals in their bulk forms have indices between 1.47 and 1.68.^43^ Moreover, the refractive index is comparable to indices of few classical high-index materials, such as silicon (Si)^44^ and titanium oxide (TiO_2_),^45,46^ as well as emerging high-index materials, such as gallium phosphide (GaP),^47^ tin(IV) sulfide (SnS_2_), and tin selendide.^48^

Although the reflectivity of Fe_Y_ of all three heterostructures is very similar to that of the pristine ones, the values of Al_Y_ show a considerable increase in the infrared and visible range for the in-plane and out-of-plane polarizations. Hence unlike Al_Y_ systems, the Fe_Y_ structures continue to be transparent in the infrared and visible range for the in-plane and out-of-plane polarizations. We observe a significant increase in reflectivity compared to lizardite and clay minerals in general, which have low reflectivity.^41^ The values in the infrared region for Al_Y_ systems are much higher than that for h-BN,^49^ demonstrating an increase in the anisotropy between the values for different polarizations. Moreover, in all of the above cases, the reflectivity for in-plane polarization is much higher than that for out-of-plane polarization. In the ultraviolet range, reflectivity increases gradually until a maximum is reached before decreasing again, leading to transparency of the structures.

EELS spectra of the heterostructures of the substituted lizardite and h-BN demonstrate an increase in the energy loss for the entire range of the energy chosen, as can be seen from the fourth row of Figures 5–7. EELS at lower energies was negligible for the pristine heterostructures.^41^ It is interesting to note that Fe_Y_ has nearly zero energy loss in the infrared region and visible range. However, Al_Y_ exhibits a higher value for this range. A strong peak at around 1.5 eV is observed in Al_Si_ for three types of architectures of the heterostructures. It suffers the maximum modification in Al_Si_ and Al_Mg_ in the infrared and visible spectrum compared to its pristine counterparts. EElS increases with energy and has a maximum at around 17 eV for all of the structures for **E** ∥ *z*, **E** ∥ *x*, and **E** ∥ *y*. Energy loss in these structures can be as high as 3 in the ultraviolet range, which was also observed in aluminum oxide (α-Al_2_O_3_) and transition metals.^50,51^ Similar features were also observed in graphene/MoS_2_ heterostructures.^52^

## Conclusions

The formation of the heterostructures from the stacking of the substituted lizardite slab and h-BN layers has been addressed using density functional theory calculations. Al and Fe impurities are selected because of their abundance in lizardites in nature. The position of the h-BN layer and the type and position of the impurity atom in lizardite lead to 12 possible conformations. We observe that the heterostructures formed from h-BN and substituted lizardite are stable, and strong doping arises from the above coupling. In the presence of impurities (Al or Fe), a significant charge transfer occurs. The modification of the electronic properties of the heterostructures due to the presence of the impurities has been confirmed by the density of states and energy band analyses. While the pristine lizardite/h-BN heterostructures are semiconductors, the addition of the impurities leads to a transition to metallic behavior due to the presence of Al. The Fe_Si_ systems maintain their semiconducting properties as the pristine case, whereas Fe_Mg_ demonstrates metallic characteristics. Optical characteristics undergo modifications compared to those of the pristine lizardite/h-BN heterostructures. The modifications in the optical properties of the systems in the infrared and visible regions of the spectrum are noteworthy. The higher refractive indices and reflectivity for Al_Si_ and Al_Mg_ are evident. Hence, the heterostructures with tunable properties could have potential applications in optoelectronic devices and field-effect devices. The ability to switch between the metallic and semiconducting states dependent on the dopant could be useful due to its conductivity, which could be controlled by the formation of the heterostructures. The experimental verification of the above results is highly necessary, although we know that difficulties could arise due to not only material synthesis and lattice mismatch but also interface quality, characterization methods, and environmental stability. However, the presence of impurities in the lizardite slab shows potential for enhancing optoelectronic device performance.
